# System Models for Synchronous Strategies in Operational Healthcare Forecasting

**DOI:** 10.3390/ijerph22020265

**Published:** 2025-02-12

**Authors:** Arnesh Telukdarie, Logistic Makoni, R. Raghunatha Sarma, Megashnee Munsamy, Sunil Kumar

**Affiliations:** 1 Johannesburg Business School, University of Johannesburg, Johannesburg 2006, South Africa; logisticm@uj.ac.za (L.M.); mmunsamy@uj.ac.za (M.M.); 2 Vidyagiri, 5R77+32M Prasanthi Nilayam, Puttaparthi 515134, Andhra Pradesh, India; rraghunathasarma@sssihl.edu.in (R.R.S.); psunilkumar@sssihl.edu.in (S.K.)

**Keywords:** healthcare delivery, healthcare forecasting, LMICs, optimization, digital tools, artificial intelligence (AI)

## Abstract

The delivery of healthcare in Low-to-Medium-Income Countries (LMICs) has long posed challenges, with established models predominantly found in wealthier nations. These models are found to be either strategic or operational, and very rarely combine these two perspectives. Most importantly, these models lack a comprehensive, holistic and synchronous construct that accompanies a systems thinking approach. This research evaluates international best practices, fundamental global theories and existing systems and tools in healthcare through a systems approach. It collates these data to propose a customized systems-based, comprehensive framework for modeling and optimizing both the management and operational tiers of healthcare in LMICs. The approach is based on the adoption of digital tools, inclusive of AI, to analyze, assimilate, align and develop advanced, holistic and inclusive frameworks. The current gap in global healthcare delivery is characterized by an ongoing lack of ability to provide quality and cost-effective care, especially in the LMICs. Despite the fact that developmental challenges are unique and specific to respective countries, there are commonalities with regard to healthcare processes that present opportunities for optimization. The main challenge lies in the effective collation and synchronization of data and tools with the specific contexts of each country. This situation highlights the need for a cohesive systems approach to enhance healthcare delivery in LMICs, allowing for tailored solutions that can bridge existing gaps. This paper presents a strategic model, with initial data quantification guiding the development of the system model. The practical significance of this research lies in its potential to transform healthcare delivery in LMICs, leading to enhanced access and quality of care through optimized systems.

## 1. Introduction

**Figure 1:**
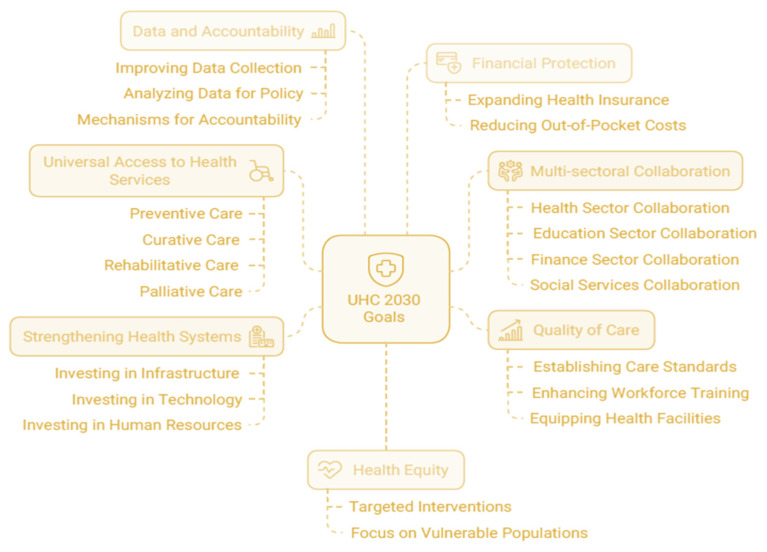
ILO universal healthcare.

The healthcare landscape in Low-to-Medium-Income Countries (LMICs) is faced with a myriad of challenges, such as lack of resources, limited infrastructure and lack of sufficient healthcare policies [1]. As per the Sustainable Development Goal (SDG) 3 targets, all countries are supposed to achieve healthy lives through appropriate strategies that promote people’s well-being at all ages for sustainable development which includes universal health coverage (UHC). COVID-19 was a stark reminder of the fact that the world is not very well prepared for a wide range of health issues which could impact humanity. Despite noteworthy improvements in global health, there remain disparities in healthcare delivery, especially when comparing LMICs to developed countries [2]. The traditional models of healthcare systems, largely influenced by the systems and abilities of high-income countries (HICs), normally overlook the unique socioeconomic and environmental situations of LMICs, which results in suboptimal health outcomes [3]. One of the main challenges experienced in LMICs is the ineffective management of healthcare resources, coupled with limited integration of data systems that can influence strategic decision-making [4]. Even though the UHC2030’s 2024–2027 Strategic Framework for universal health coverage describes strategies such as advocacy, accountability and alignment for a transformation to follow in 2024–27, problems such as financial constraints and a lack of efficient frameworks that can cater for the intricacies of health needs trigger disparities in healthcare delivery systems [1,4]. Figure 1 depicts the goals of UHC envisioned by the ILO.

As disclosed in the [5] report, healthcare systems must have efficient investments that are supported by models and frameworks that make the provision of effective resource allocation and usage possible, as well as monitoring and evaluation of associated processes. To achieve sustainable healthcare provision, it is vital to align operational processes with the strategic goals of the healthcare sector [4].

Today’s digital landscape is characterized by transformative digital technologies, such as artificial intelligence (AI), that have emerged as innovative tools for the healthcare sector [6]. These tools have the potential to streamline operations, while simultaneously facilitating real-time decision-making and improving data management. For example, healthcare providers can forecast and optimize resource allocation and operations through employing AI-driven analytics [7]. For this to happen in LMICs, there is a crucial need for a meticulous consideration of socioeconomic and other related developmental challenges by healthcare stakeholders, policymakers and developers, which is currently a huge omission in this context [8].

The main objective of this research is to close this gap through the development of a comprehensive system model for synchronous strategies in operational healthcare forecasting in the context of LMICs. This model is anticipated to serve as a framework for the modeling and optimization of healthcare management and operations in LMICs. Through the evaluation of global best practices and significant global theories in healthcare, this research aims to provide substantial insights into the unique opportunities and challenges faced. In addition, this research places emphasis on the significance of the systems approach that integrates strategic initiatives with operational efficiencies, ultimately bridging the gap in healthcare delivery.

The existing gaps in healthcare delivery, normally associated with insufficient provision of effective care, underscore the urgency of this research. Essentially, there is a need to learn from best practice though a systems approach, and construct and use the very same construct (model) to guide current strategies. More especially, the myriad of challenges faced in LMICs requires the construction of tailored solutions though a comprehensive framework for healthcare systems; this is crucial for defining common processes that can be implemented across the respective countries to enhance their healthcare sectors. This research contributes to the ongoing discourse regarding healthcare optimization in LMICs, providing both theoretical and practical implications that can drive meaningful changes in health systems.

## 2. Literature Review

### 2.1. Defining Healthcare

Healthcare is a multidisciplinary field that incorporates the diagnosis, prevention, treatment and management of diseases and injuries. It involves health and well-being. The aim of healthcare is to improve people’s and communities’ quality of life through providing medical services, as well as various measures of disease and injury prevention. The earliest definition of healthcare identifies the concept as a “state of complete physical, mental, and social well-being” [9]. In this definition, [9] places emphasis on the need to address many issues regarding people’s health, such as mental, physical and social aspects. In its broad sense, healthcare involves a comprehensive approach to improving quality of life and is not limited to injuries and/or diseases.

**Figure 2:**
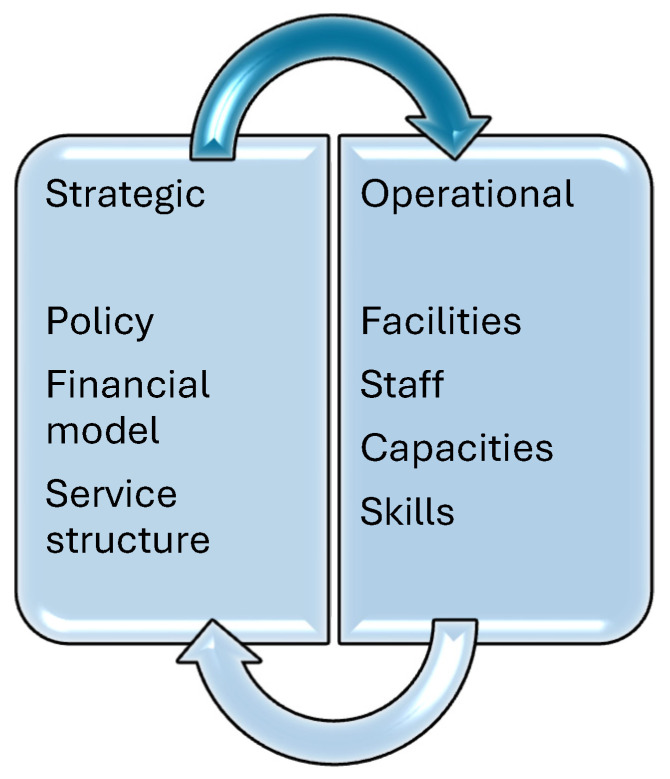
Levels of healthcare.

The concept of healthcare delivery, in this sense, comprises a system of ample inter-related elements that work together to fulfill the health needs of individuals and communities. These elements include several factors that can shape an integrated network, such as healthcare facilities and providers, policies and regulations, and technology. According to [10], efficient healthcare requires a combination of stakeholders and services across various levels to ensure that swift and appropriate care is given to people. This flexibility is important, especially in the case of LMICs, where limitations in terms of resources and many health challenges require tailored solutions [2]. Healthcare delivery operates on two key levels: strategic and operational. Figure 2 presents these two levels.

Figure 2 portrays how the healthcare system in any country can be conceptualized as operating on two key layers, namely the strategic layer and the operational layer [11]. The strategic layer involves high-level decision-making, resource allocation, and the development of long-term plans to improve population health outcomes. It is driven by various factors, including national legislation, prevailing economic conditions and types of funding mechanisms, as well as global and local policies. These various factors work together to shape the overall direction and priorities of the healthcare system.

**Figure 3:**
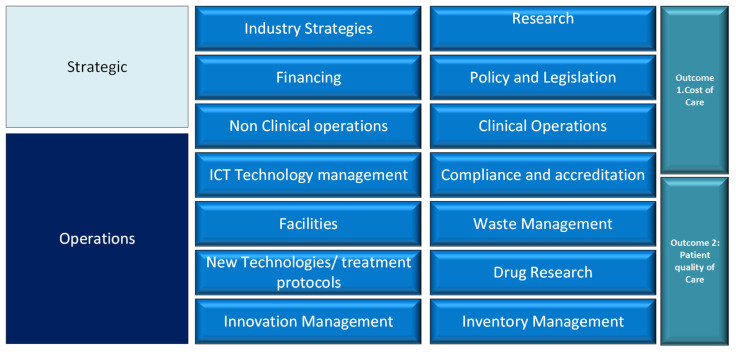
Comprehensive overview of healthcare delivery.

While the strategic layer sets the framework within which healthcare services are delivered, the operational layer focuses on the implementation of these strategies at the facility level. This layer deals with the day-to-day functioning of healthcare facilities, including patient flow management, staff scheduling and the procurement and maintenance of medical equipment and supplies [11,12]. Figure 3 provides a comprehensive insight into the two levels of healthcare delivery.

Effective healthcare delivery hinges upon the perfect alignment of, and synergy between, the strategic and operational layers. However, there is a gap in how health strategies are currently implemented at the operational level, where the day-to-day functioning of healthcare facilities occurs [13]. Consequently, operational inefficiencies, such as poor inventory management and suboptimal clinical protocols, are common in LMICs, contributing to the generally poor performance of their healthcare systems [14,15].

These two levels must work together to create an efficient healthcare system that can effectively respond to the needs of the community while ensuring that strategic goals are met [16]. This dual approach highlights the importance of both planning and execution in the quest for effective healthcare delivery, especially in LMICs, where this is lacking. The literature on healthcare challenges in LMICs broadly highlights various barriers to effective healthcare delivery, but there is a need for more in-depth exploration of the specific contextual challenges faced by these countries. One critical factor that is often underexplored is the role of sociopolitical changes in shaping healthcare infrastructure and access. Political instability, frequent changes in government, corruption and weak governance structures are common in many LMICs, and can significantly undermine efforts to build and maintain healthcare systems. Such changes often lead to poor policy implementation, misallocation of resources and disruptions in healthcare services, which disproportionately affect vulnerable populations. Furthermore, the interplay between sociopolitical changes and economic constraints often exacerbates the strain on healthcare systems, hindering the development of sustainable and effective healthcare solutions. While the existing literature acknowledges these issues, a more nuanced analysis of their impact on healthcare infrastructure and service delivery is needed to fully understand the complexities that LMICs face in achieving health system improvements.

Sociopolitical problems normally result in significant disruptions in the healthcare sector in LMICs, due to the existing corruption and governance issues. For example, in South Sudan, where there is political unrest, the healthcare infrastructure is usually ignored or damaged, which makes it difficult for people to have normal access to healthcare services. Also, in the case of Venezuela, where political instabilities have been prolonged, affecting economy stability, healthcare challenges have not been immune to this situation. It is difficult to provide basic healthcare for people under the current situation.

### 2.2. Need for Tailored Healthcare Models for LMICs

LMICs have often attempted to adopt the healthcare models from higher-income countries, but without adequately contextualizing them to local conditions [17,18,19,20]. This lack of adaptation has hindered the delivery of accessible, affordable and high-quality care, leading to operational inefficiencies, and resulting in disparities in health outcomes when compared with high-income countries [20]. To bridge this gap, it is imperative to move beyond the mere replication of high-income country models, and embrace a systems approach that accounts for the unique context of LMICs [19]. This approach recognizes the complex interplay of factors influencing healthcare delivery, and seeks to optimize the system as a whole, rather than focusing on isolated components [21].

### 2.3. Role of Digital Technologies in Healthcare Optimization

The adoption of the Fourth Industrial Revolution (4IR) tools, including artificial intelligence (AI) and machine learning (ML), offers significant opportunities for optimizing healthcare delivery in LMICs. AI and big data analytics have been used to streamline operations, improve decision-making, and enhance patient outcomes [22]. Machine learning algorithms have facilitated the analysis of large health datasets to identify patterns and predict outcomes, allowing for more proactive and efficient healthcare management [23]. Leveraging AI for diagnostic protocols and patient monitoring can enhance the efficiency of healthcare delivery, especially in high-patient-load situations within resource-limited environments [24].

Despite these positives, however, there exist challenges associated with implementing these technologies in LMICs, such as a lack of digital infrastructure, data quality issues, regulatory obstacles, the need for ethical and social considerations and the need for contextual adaptation [15,19,25]. This indicates that while digital technologies are available, their deployment is often impeded by systemic issues within LMICs’ healthcare infrastructure. The solution to this problem is to devise strategies to overcome the identified barriers to digital implementation. For instance, developing robust eHealth environments, ensuring the involvement of local stakeholders in AI projects, and creating standardized guidelines for AI applications in healthcare. These strategies aim to foster an ecosystem that is conducive to the successful integration of AI technologies in LMICs [19,24].

Business process modeling (BPM) is one such tool, whose use is gaining traction in healthcare optimization. BPM can facilitate the identification of bottlenecks, inefficiencies and opportunities for improvement through mapping out processes within a healthcare facility [26]. Moreover, the analytical capabilities of BPM are further enhanced by integration with digitals tools such as AI and Business Intelligence (BI) [27]. For example, while BPM can be a valuable tool for visually representing and analyzing the workflows and activities within a healthcare system, AI can be leveraged to automate data collection and analysis, facilitating the identification of patterns and trends, and the generation of insights for decision-making [28,29]. There are positive outcomes from the application of BPM and digital tools in healthcare settings, including reduced waiting times, improved resource utilization and enhanced patient satisfaction [29,30].

The concept of digital twins, which are virtual replicas of physical systems, has also been applied to healthcare, allowing for real-time monitoring and optimization of healthcare processes [31]. The literature suggests that combining BPM with digital twins can significantly enhance the ability of healthcare systems in LMICs to adapt to changing conditions and improve overall performance [32]. Furthermore, the integration of digital twins with BPM tools can provide real-time visualizations through dashboards, facilitating the monitoring and evaluation of healthcare processes [33].

The incorporation of expert systems, a branch of AI that emulates the decision-making ability of a human expert, can further augment healthcare optimization efforts. Expert systems leverage a knowledge base and inference engine to simulate the decision-making capabilities of human experts [34]. This knowledge base is populated with domain-specific information, encompassing medical guidelines, clinical data, and expert insights [35]. The inference engine applies logical rules to this knowledge base in order to generate conclusions or recommendations, aiding healthcare professionals in diagnosis, treatment planning and decision support [35].

Expert systems have a demonstrable history of successful application in healthcare optimization, especially in clinical decision support and diagnostic assistance, where their ability to learn from accumulating data improves their accuracy and reliability over time [36,37]. This is particularly pertinent in LMICs, where a shortage of specialized healthcare professionals can limit access to quality care [37]. By simulating expert judgment, these systems can assist healthcare providers in making informed choices even in resource-constrained settings, thereby bridging gaps in expertise and enhancing care delivery [37].

### 2.4. Role of Modeling and Forecasting in Healthcare Optimization

The application of modeling and forecasting techniques can enable proactive healthcare management in LMICs, especially when driven by AI and big data analytics. Predictive models can be developed to anticipate disease outbreaks, estimate resource needs and identify high-risk populations, allowing for targeted interventions and facilitating efficient resource allocation [38]. Simulation models can be used to test the impact of different policies and interventions, facilitating evidence-based decision-making and resource optimization [39]. For example, [38] developed a predictive model using AI algorithms and real-time data to forecast infectious disease outbreaks, allowing for timely containment measures and effective resource distribution. Similarly, [38] demonstrated the value of simulation modeling in optimizing healthcare policies to improve patient outcomes and resource use in LMICs.

The literature emphasizes the importance of integrating modeling and forecasting into healthcare planning and management to improve preparedness, responsiveness, and overall system resilience [40]. However, successful implementation requires addressing challenges such as data scarcity, limited technical expertise and inadequate infrastructure. Investments in data systems, capacity building and technology can unlock the full potential of these techniques to strengthen healthcare systems in LMICs [38].

### 2.5. Best Practices for Healthcare Services: Digital Technologies, Modeling and Forecasting

Adopting global best practices in healthcare is essential for improving the quality of care in LMICs. Successful implementations of global best practices in LMICs have led to improvements in maternal and child health, infectious disease control and overall health system performance [18,41,42]. Adoption of best practices can accelerate progress, with technology playing a crucial role in facilitating the dissemination and adoption of best practices, and enabling LMICs to leapfrog traditional development pathways [43].

However, while the adoption of global best practices is essential, it is equally important to adapt these practices to local contexts to ensure their effectiveness [44]. Some of the AI applications developed in high-income countries may not be directly applicable to LMIC contexts. The literature underscores the need for a balanced approach that leverages global knowledge while considering local realities, such as resource constraints and cultural factors [45]. Access to information and knowledge regarding successful interventions and strategies implemented elsewhere can inform decision-making and guide the adaptation of these practices to local conditions, with previous studies indicating that healthcare interventions are more successful when they are tailored to the specific needs and conditions of the local population [46,47].

There is a multifaceted nature to healthcare challenges and opportunities in LMICs, and there is a need for a holistic approach that considers the interplay of strategic, operational and contextual factors. While global best practices and advanced digital tools offer significant potential for improvement, the success of these interventions depends on their adaptation to local contexts. A systems thinking approach, combined with the use of AI, BPM and digital twins, provides a comprehensive framework for optimizing healthcare systems in LMICs.

The proposed healthcare optimizer platform, grounded in systems thinking, global best practices, business process mapping and digital tools, offers a promising avenue for addressing these challenges. By enabling the contextualization and adaptation of best practices, facilitating data-driven decision-making and optimizing healthcare processes, this platform has the potential to significantly improve healthcare delivery and outcomes in LMICs. This research, therefore, focuses on the development and implementation of such a platform, evaluating its effectiveness in diverse LMIC settings.

### 2.6. Research Grounding Theories and Models

The development of AI-driven optimization tools for healthcare systems in LMICs necessitates a strong theoretical foundation to ensure their effectiveness and sustainability. As both the strategic and operational layers are subject to dynamic internal and external influences, a systems thinking approach that acknowledges the interconnectedness and adaptability of the healthcare system is essential [48].

#### 2.6.1. Systems Thinking

Rooted in the General Systems Theory proposed by [49], systems thinking provides a holistic approach that considers the system as an integrated whole, characterized by complex interactions among its components, feedback loops and emergent properties [50]. This holistic perspective shifts focus from individual components to the system’s overall structure, behavior and interdependencies [51].

Systems thinking is increasingly recognized as a crucial approach to addressing the complexities of healthcare systems [52,53]. This is one of the major gaps identified in this study—that healthcare systems in LMICs are often considered separately rather than as a whole, which affects the strategic and operational approach overall. The integration of systems thinking, therefore, could foster more holistic and effective solutions in these settings.

Systems thinking enables a deeper comprehension of the factors influencing health outcomes, aiding in the identification of points for intervention and optimization [54]. Research has demonstrated the value of systems thinking in addressing complex healthcare challenges, particularly in LMICs [55,56,57]. It has been applied to diverse areas, such as health financing, human resources for health and service delivery, contributing to improved policy formulation, resource allocation and overall system performance [58,59]. Several other fundamental theories collectively complement systems thinking to provide a strong foundation for the design and implementation of AI-driven healthcare optimization tools tailored to the specific needs of LMICs:

- Complexity Theory recognizes the unpredictable nature of healthcare systems, where AI can manage complexity by detecting patterns and predicting outcomes from vast datasets [60]. By leveraging machine learning algorithms, AI tools can adapt to changing conditions in LMICs, providing insights that support proactive healthcare management [37].
- Diffusion of Innovations Theory, developed by Everett Rogers in 1962, explains how new technologies spread within a society [61]. In the context of LMICs, strategies for successful implementation and AI adoption are influenced by factors such as perceived benefits, compatibility with existing practices and supportive policies [62].
- Health Systems Strengthening Frameworks focus on improving the performance of health system components, including service delivery, health workforce, information systems and governance [63]. AI can support these components by providing data-driven insights, tailored to LMIC contexts, that help to improve service delivery efficiency and resource allocation [64]. The emphasis on context-specific solutions within these frameworks makes them particularly relevant to LMICs [64].
- Behavioral Economics can assist in examining how psychological, cognitive, emotional, cultural and social factors influence decision-making in healthcare [65]. Understanding these factors can help in the design of AI tools that align with the behaviors and preferences of healthcare providers and patients in LMICs [66].

Collectively, these theories can support the development of AI tools that are contextually relevant, technologically advanced and user-centric, addressing the unique challenges in LMIC healthcare systems and promoting equitable care access. Four globally well-established financial models for an NHS are identified:

#### 2.6.2. Beveridge Model

The Beveridge model is a healthcare system designed to cater to the healthcare needs of all the citizens of the country. This system is run with funds collected through taxation, and it is the government’s responsibility to allocate necessary services and funding to make sure that all healthcare resources are in place. This healthcare cover is accessible to all citizens regardless of financial status, from deprived to affluent citizens. Equality of access to healthcare should be achieved independently of a person’s ability to pay, or of other factors like income and place of residence, in a real Beveridge-model public system. A national health service and universal coverage are examples of systems framed under the Beveridge model; they offer healthcare services to all to offload the financial burden from less fortunate citizens who cannot afford healthcare needs.

#### 2.6.3. Bismarck Model

The Bismarck model is a healthcare system funded through mandatory insurance contributions by both employers and employees. Even though it is an insurance-based healthcare system, it caters for all citizens, but with the help of government subsidies. Making healthcare affordable for those who cannot otherwise afford treatment is a top priority for high-income nations. To ensure that lower-income people and families can access care without fear of suffering a catastrophic financial loss, common strategies include paying for the care of population groups in need, lowering patient cost sharing requirements, tying out-of-pocket expenses to income or covering additional services like dental care. Patients have a choice to use private or public hospital healthcare services.

#### 2.6.4. Out-of-Pocket

**Figure 4:**
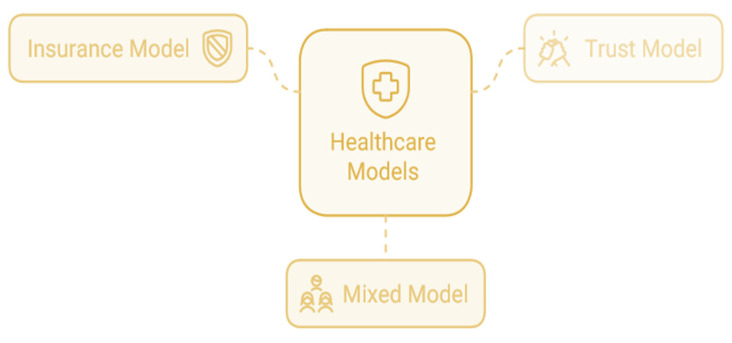
OOP model.

Out-of-pocket expenses are healthcare costs paid towards healthcare services; these expenses are paid directly, without the involvement of any healthcare insurance borne by the beneficiary. One of the primary methods of paying healthcare in many nations is through out-of-pocket (OOP) payments. An OOP model can consist of any one or a combination of three models, as shown in Figure 4.

Paying for medical services out-of-pocket guarantees instant cash flow into the healthcare system, which is especially beneficial in underfunded healthcare settings, where insurance companies or the government may take longer to pay healthcare providers. The In-Patient OOP model [67] basically deals with the package components of the hospitalization costs, doctor’s fees, diagnostics and medicine costs during hospitalization, and excludes any amount reimbursed by insurance. Flexibility is given to the concerned health authorities to choose between two service purchase or provider models: the Insurance model, where an insurance company is contracted with annual premiums, bearing financial risk while sharing excess costs; and the Trust model, where a state-run trust directly buys services from healthcare providers, often supported by implementation agencies. Most states use the Trust model, while a few employ the Insurance model [68].

#### 2.6.5. Hybrid Models

A hybrid model is a mix of the above models. These financial models have been adopted in various countries with varying degrees of success. It is important to note that the selection of the financial model is based on the history and the current and future trajectory of the NHS; constraints on costs, as demanded by the insurer or trust for the service; and the SOP, agreed upon between the concerned health agency and the service provider. Thus, it is not based on actual data on the current operations of the NHS.

### 2.7. Summation and Review of Research Opportunities

The literature review highlights the multifaceted nature of healthcare challenges and opportunities in LMICs, and underscores the need for a holistic approach that considers the interplay of strategic, operational and contextual factors. While global best practices and advanced digital tools offer significant potential for improvement, the success of these interventions depends on their adaptation to local contexts. A systems thinking approach, combined with the use of AI, BPM and digital twins, provides a comprehensive framework for optimizing healthcare systems in LMICs.

The proposed healthcare optimizer platform, grounded in systems thinking, global best practices, business process mapping and digital tools, offers a promising avenue for addressing these challenges. By enabling the contextualization and adaptation of best practices, facilitating data-driven decision-making and optimizing healthcare processes, this platform has the potential to significantly improve healthcare delivery and outcomes in LMICs. This research therefore focuses on the development and implementation of such a platform, evaluating its effectiveness in diverse LMIC settings.

Overall, despite the literature providing practical insights into the need for customized healthcare models for LMICs, together with the significance of systems thinking and the role of digital technologies, there remain several challenges and gaps that need to be addressed. For instance, limited attention in research is given to the integration of digital technologies and modeling in healthcare forecasting in the context of LMICs, despite the existence of literature on the significance of these technologies and models in healthcare optimization. In addition, much of the existing literature focuses largely on theoretical overviews and models, with limited pragmatic executions and real-world case scenarios, especially in resource-constrained contexts. Further, the need for more research on how to effectively integrate digital technologies with traditional healthcare infrastructure in LMICs is vital. Also, while systems thinking is constantly mentioned, its application in bridging strategic planning with operational healthcare forecasting remains underexplored. Thus, a significant research opportunity exists of developing a model that links strategic to operational healthcare forecasting, specifically one that is tailored to the unique challenges of LMICs. Such is the purpose of this research. The system model assists in achieving this purpose.

The systems thinking model is central to this research, because it aligns with the objective of the design of a comprehensive and synchronized system model for healthcare forecasting in LMICs. Through looking at the healthcare systems comprehensively, systems thinking helps in identifying the interdependencies between the strategic and operational layers. This helps in ensuring that these layers are balanced effectively. In addition, the systems thinking approach puts emphasis on the need for understanding how various elements [such as finances, infrastructure and policies] influence one another within the healthcare system. Regarding this research, applying the systems thinking approach aids the goal of the optimization of the healthcare sector by providing tailored solutions that address the unique challenges of LMICs, while simultaneously ensuring the synchronization of strategic objectives with operational realities.

## 3. Methodology

### 3.1. Theoretical Frameworks Guiding Methodological Procedures

**Figure 5:**
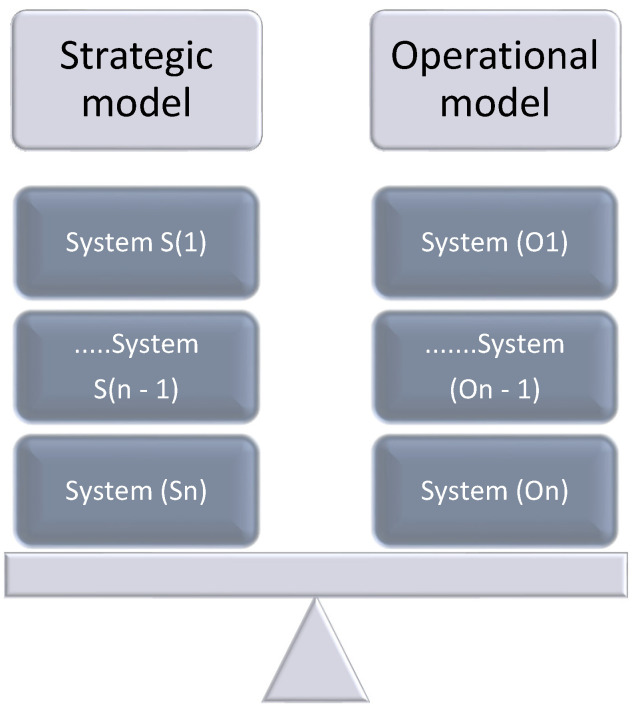
The two layers of strategic and operational models.

The authors assume a systems approach [52,53] where synchronization of the strategic and operational layers is essential. The approach includes the development of models for the strategic layer and the operational layers, with the ability to manage the synergies and achieve bi-directional balance, as portrayed in Figure 5.

The two layers require significantly diverse approaches, as their constructs differ.

### 3.2. Strategic Modeling

The strategic layer is grounded in the four healthcare models discussed in the Literature Review Section of the paper.

**Figure 6:**
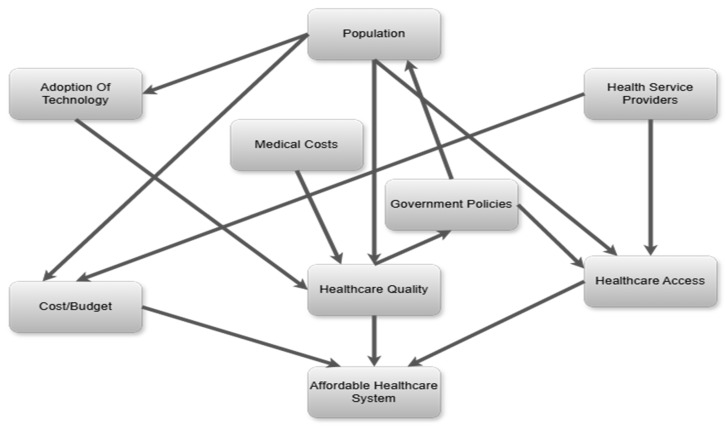
Structural representation of critical factors.

The research team approached the strategic layer with systems paradigms, attempting to collate a global perspective on each of the four models. A multi-stage, best practice, qualitative to quantitative analysis was undertaken. The qualitative to quantitative analysis began with a review of the financial models to identify key variables. Each fundamental healthcare model has its own diverse unique capabilities for ensuring better-quality healthcare; the difference between each model is based on numerous factors that impact its performance. These differences are discussed and understood but need further analysis as part of the research and interpretation of the results to support the findings of each model. Data analysis can not only provide support for the findings, but also unearth unknown discoveries that are useful in understanding each system in depth. Healthcare systems are diverse and complex, and each is designed to serve a specific purpose. The team commenced with expert knowledge development through an initial literature review, as illustrated in Figure 6.

To better understand the performance behavior of each healthcare model, the systems thinking model can be implemented. Systems thinking necessitates that a system— in this case, the national healthcare system (NHS)—is considered holistically with feedback loops, as a system is greater than its individual parts, and results in emergent behaviors. This is especially relevant, as there are numerous stakeholders in the NHS, with their relations and interactions continuously evolving and non-linear. The NHS variables can be considered as the external environment, internal stimuli and external stimuli.

**Figure 7:**
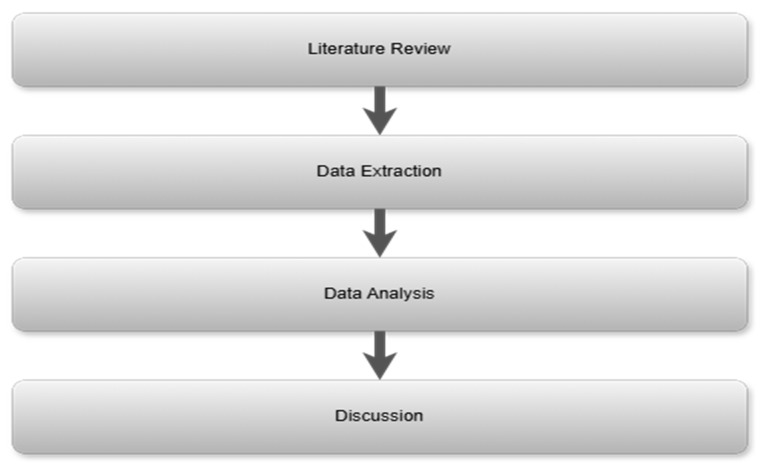
Methodology structure.

Comparative analysis of secondary data, such as academic literature from Scopus and ScienceDirect, policy documents and reports from international health organizations, was used in this study. The goal was to offer a thorough overview of the various methods of healthcare financing and service delivery by looking at the government policy frameworks and healthcare models in each nation. To evaluate the efficiency of each model, important metrics like healthcare costs, population and healthcare infrastructure are part of the study. Figure 7 below presents the methodological structure employed in this research.

- Data sources from literature review

In the research, the information was sourced and insights on healthcare systems were gathered. Academic papers were collected to unpack the performance of each healthcare system. Differences between private and public healthcare were revised, with a focus on comparing their benefits, services and infrastructural disparities. The costs of private care services and funding injections towards public care were also uncovered. Finally, the critical factors influencing the performance of each healthcare system, along with their challenges and advantages, were examined.

- Data extraction

The data extraction process for this research involved gathering relevant information using a set of carefully chosen search strings, as shown below:

NHI (National Health Insurance), NHS (National Health Service), UHI (Universal Health Insurance), UHC (Universal Health Coverage), NHIF (National Hospital Insurance Fund), GHC (Global Health Coverage), HIP (Health Insurance Program), HPG (Healthcare Policy and Governance), HEA (Healthcare Equity and Access), HCC (Healthcare Cost Containment), PHS (Public Health System), SPS (Single-Payer System), MPS (Multi-Payer System), HFM (Healthcare Financing Model), HR (Healthcare Reform), SHIS (Social Health Insurance System), HQA (Healthcare Quality Assurance), HFS (Healthcare Financing Strategy), Telemedicine (Telemedicine), PHC (Primary Healthcare), ACO (Accountable Care Organization), PPO (Preferred Provider Organization), HMO (Health Maintenance Organization), POS (Point-of-Service), VBPM (Value-Based Payment Model), IDS (Integrated Delivery System), PCMH (Patient-Centered Medical Home), DPC (Direct Primary Care), SIB (Social Impact Bond), Crowdfunding (Crowdfunding), Microlending (Microlending), CHNA (Community Health Needs Assessment), SDOH (Social Determinants of Health), GHI (Global Health Initiative), SHIRE (Social Health Insurance with Risk Equalization), CBHI (Community-Based Health Insurance), PPP (Public–Private Partnerships), HAS (Health Savings Account), FSA (Flexible Spending Account), ACO (Accountable Care Organization), Assupol, (Assupol), Discovery Health (Discovery Health), MMH (Momentum Metropolitan Health), LH (Liberty Health), Medihelp (Medihelp), BMA (Bonitas Medical Aid), FMA (Fedhealth Medical Aid), GEMS (Government Employees Medical Scheme), HMA, (Hearth Medical Aid), RH (Resolution Health), KH (KeyHealth), CMS (Council for Medical Schemes), RAND HIE (RAND Health Insurance experiment).

Applying a named entity recognition model to extract text to identify and classify named entities, this tool’s purpose is to express structured data in a machine-readable format by extracting it from unstructured text data. Approaches generally employ BIO notation, which distinguishes between an entity’s inner (I) and beginning (B). For non-entity tokens, O is utilized. A number of critical factors were identified from named entity recognition for further analysis.

- Data Analysis

The data gathered from the papers helped to understand these factors’ relationships and how they either directly or indirectly affect one another. Using the word2Vector model to further investigate the word frequencies from the papers, this approach helps with obtaining word counts and the semantic proximity of words, helps in understanding the contextual relationships between words, in order to identify and provide insight into trends that might not be evident at first glance.

### 3.3. Operational Modeling

The operational models would be approached with systems thinking, in a structured and quantitative manner. This would include a structured analysis of facilities through activities analysis, as captured with business processes, and a critical analysis of data and systems to secure sufficient insights into country-specific operations.

Business processes detail the execution of business activities. These activities range from management, to support, to operational. Typically, business processes are hierarchically structured, are finite, and illustrate interlinkages and inter-relationships, functionally and cross-functionally, which are captured across all levels of hierarchy. Hence, business process mapping enables a comprehensive representation of a business. BPs are data centric; the more information available, the better the decision-making.

**Figure 8:**
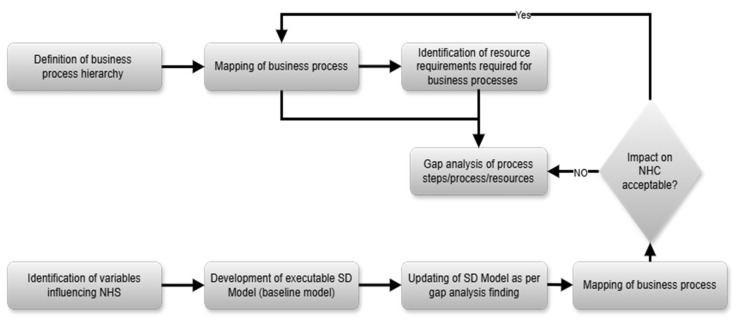
Methodology for the operational layers.

With the concepts of system thinking and business process mapping, a mixed method approach is used to develop the NHS optimization platform, as illustrated in Figure 8.

Figure 8 illustrates the back end of the NHS optimizer. A front-end user interface will be developed to enable the user to optimize the NHS. The research procedures employed in this study, as discussed above, were informed by the literature and theoretical constructs.

## 4. Results

Based on the conducted literature, there is inequality in terms of good-quality healthcare services in LMICs. Insufficient healthcare services for deprived citizens have been an issue in most countries in the region, and each country strives to address such a problem. The goal of this study was to comprehend the healthcare system as a whole, to identify the variables that impact performance and to develop a more effective strategy mitigation to enhance the system. Initially, the researchers collected 1000 papers for the research. These papers were generated through the main keywords search for “Healthcare” and “healthcare models”. The four fundamental theories discussed under the results of this paper—Bismarck, Beveridge, Out-of-Pocket and Hybrid—were found to be the common financial theories emerging in all of the searched papers. These were found to be the basic strategies and models across the healthcare spectrum.

### 4.1. Strategic Model Analysis

Each of these models has a structure designed to address particular goals. The researchers further gathered 1500 papers for each model, and ran them through Word2Vector and heatmapping. After investigating each model, the researchers discovered that none of them is flawless; each has drawbacks of its own, and resolving these problems requires a deeper comprehension of the system as a whole and its shortcomings. The analysis of each model is presented below.

### 4.2. Beveridge System Analysis

Analyzing the Beveridge model to uncover and understand the driving forces, limitations and challenges in graphical respresentation, the following analyses are intepreted graphically.

**Figure 9:**
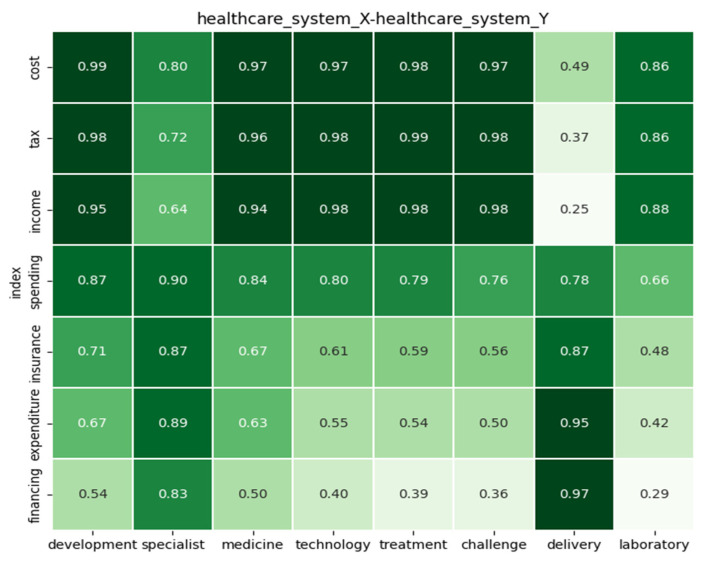
Analysis of factors under Beveridge model.

Looking at the above Beveridge model statistics, it is understood that finance is one of the driving forces supporting the model system. The Beveridge model is funded through tax collections and other donations from external sources; the relation between tax and development, medicine, technology and treatment supports this statement, since the relational strength is very high for these factors. In the Beveridge system model, one of the challenges affecting the performance of the healthcare system is healthcare skills, doctors, nurses or healthcare providers. Figure 9 illustrates the relation rate between tax and specialist (as healthcare providers), showing a rate just above the average overall rate, giving an evident reason for the lack of healthcare providers.

The healthcare system is for all citizens, regardless of financial status, and it is the government’s responsibility to make sure that public hospitals have proper technology and high-quality healthcare resources. The relation between tax and delivery makes a difference in the development of the system. Tax and challenge rates are very high, indicating that there may be issues of tax collection and tax money management affecting healthcare systems. This finding is very critical, exposing the mismanagement of funds, which results in low-quality healthcare treatment, a shortage of healthcare providers and many other factors.

One of the most critical relations is population and cancer; it is well known that public hospitals do not cover most healthcare issues, and cancer is one of the very critical sicknesses that public hospitals do not fully cover. Only citizens who can afford private services can cover themselves for this type of critical issue, which shows the imbalance between private and public hospitals. The objective of NHI is to bridge the gap of inequality and strive for equal quality of treatment for all citizens, regardless of financial status. There are interesting relations between delivery and performance quality; this supports the above-stated statements regarding public hospitals and deliveries and the mismanagement of tax funds for the healthcare system. The demand for medicine, laboratories and treatment rates is very high, indicating why public hospitals cannot cover most critical illnesses. Management and delivery indicate that there are challenges regarding healthcare delivery; this can be supported by the relation between healthcare access and delivery, resulting in a high rate for challenge versus healthcare access.

### 4.3. Bismarck System Analysis

The following analysis is based on the Bismarck model. The Bismarck model is funded through employees’ and employers’ contributions. The government assists with subsidies to offload financial burdens from citizens.

**Figure 10:**
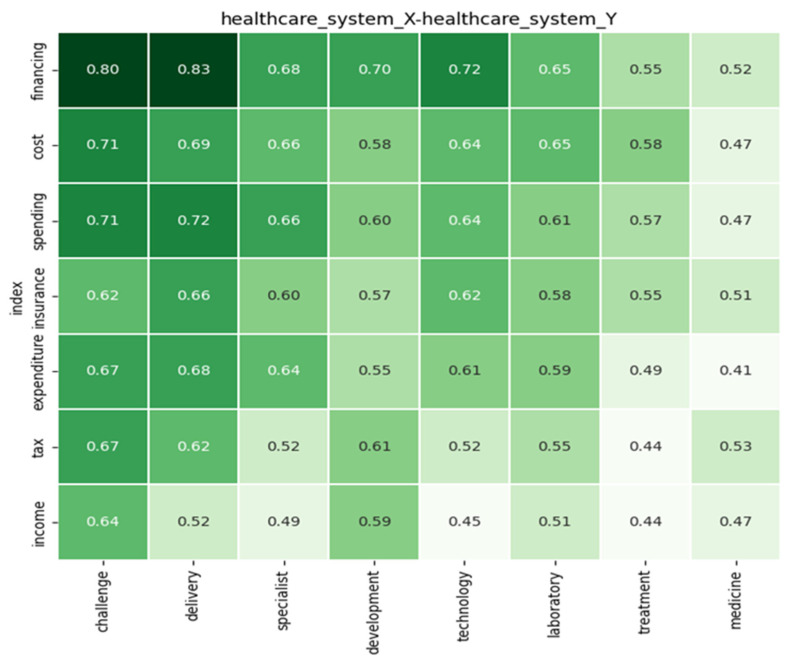
The analysis of factors under the Bismarck model.

Figure 10 indicates that the delivery and tax rates are low; this supports and emphasizes the above statement that the government will only subsidize those who cannot afford full payments for healthcare services. This system is funded through insurance. Its purpose is to cater for employees and employers; both parties contribute a margin of their income into the insurance scheme. Looking at the relationship of financing to all respective factors on X-axis, a high strength of relation is indicated, indicating the driving forces of the Bismarck healthcare system.

The figure supports the above statements by showing a high correlation between the male population and income. This indicates that the male working population enrolls in medical schemes at workplaces. Additionally, the migration rate and income suggest that most immigrants have adopted the Bismarck system to fund their healthcare needs.

Most companies with immigrants support their employees and include medical aid as part of their benefits. Since the system is supported by the contribution of employees and employers contributing to the healthcare scheme, this system depends on the wage threshold. This poses a challenge in that not all healthcare issues are covered by the healthcare system; like the Beveridge model, this creates a division between deprived citizens and affluent citizens.

There is a challenge regarding management as well; since the system is supported by the working class, this poses a challenge to the financing of the system. The perfomance of laboratories is high, but the relation of laboratories with access rates is low, providing a clear understanding that even though medical schemes pay the costs of healthcare, not everything is covered. There are particular tests that are covered by medical aid. The lack of laboratories results in some healthcare issues not being covered, or not being affordable at all to citizens with less income, since the financing depends on the margin of contributions.

Looking at the list of healthcare issues, what the Bismarck model covers depends on affordability, the relation between (specialist) healthcare providers and performance rates that are higher than the Beveridge model, indicating the improvement and the assurance of accessibility to healthcare services which are paid for; yet, the issue of inequality in healthcare accessibility poses a threat to the high cost of critical care. Only few can have access the full cover available in high-earning countries and to affluent patients, and this leaves deprived patients struggling to obtain the same healthcare treatment. These are the limitations of using the Bismarck model.

### 4.4. Out-of-Pocket Analysis

Out-of-pocket costs are healthcare costs paid towards healthcare services. These expenses are directly paid, without the involvement of any healthcare insurance. One of the primary methods of paying for healthcare in many nations is out-of-pocket payments. Paying for medical services out-of-pocket guarantees instant cash flow into the healthcare system, which is especially beneficial in underfunded healthcare settings, where insurance companies or the government may take longer to pay healthcare providers.

The out-of-pocket model is independent; no funds are injected beforehand, and no inusrance schemes are in support of any patients. Patients pay out of their own pocket to obtain full services. This has its own limitaions and advantages. Only the affluent can afford to access services, though the advantage of this is there is no waiting period. The collected healthcare costs are immediately put on a cycle to cover the healthcare infrastructure as well as covering the healthcare providers.

This statement can be supported by looking at the relation between quality and delivery. Also, for technology and quality, the rate is impressively high, indicating that the objective of healthcare providers is to provide high healthcare quality to their patients. This includes the high rate between the laboratory and perfomance, emphasizing that the financial management is well controlled and put into places where it is most needed to improve the quality of service delivery.

Good financial management is key to develop and improve quality, and for this to be achieved, proper research needs to be carried out; thus, there is a high rate for research and development, including research and technology. Looking at management’s y values, the rate is high across 80% of its relative factors, supporting the above statements.

During the phase of the pandemic, lockdowns were imposed on every citizen, including foreigners, in different countries. Most foreigners had no medical aid, and could pay for their healthcare needs out-of-pocket, since not all medical aids are supported in other countries; thus, there is a high rate for migration and pandemic. The average rates for cancer and all patients indicate the proportion of the population that can afford treatment for this critical illness; most medical insurances and public healthcare system have limited cover when it comes to critical illnesses, and most patients cover the costs out-of-pocket to obtain the best-quality care.

Another critical illness is diabetes. This disease has a high rate for most y value factors, indicating that the out-of-pocket system is highly considered, and this can only be because the rate of the population having health insurance is low; this leaves most of the population opting for public hospital services. Only when the illness is very critical do people pay to receive the best-quality treatments and services. The high value for transplants also supports the statement that only the out-of-pocket system is considered when illnesses are critical, and the expectation of high-quality services. The high rate on tax and migration shows that in the case of migration and foreigners, only two systems are highly in use: the public healthcare system, financed by through taxation, and the out-of-pocket system. This emphasizes the statement that not all medical aid can cover patients in different countries; it can only cover patients in native countries.

The issue with the system is inequality. Most governments are trying to close this huge gap by implementing healthcare models that serve patients with equal services. The quality of healthcare services is being prioritized. All the factors should be considered critical and taken into consideration to achieve a better healthcare system model. For the Beveridge model, its major objective is to target the inequality, affordability and inaccessibility of healthcare to deprived populations. Bismarck’s focal objective is to serve employees with healthcare cover. The unique qualities of each model make a huge difference in terms of model performance.

## 5. Discussion

The three fundamental models discussed in this paper—Beveridge, Bismarck and Out-of-Pocket—are representative of the three substantial healthcare frameworks, each with different characteristics and effects. The Beveridge model, which is funded by income tax deductions, emphasizes healthcare systems that are fully provided by the government, and the government is a sole provider of healthcare to the broader population of its citizens. This model provides a framework that ensures that all citizens can have full access to the available healthcare services from the government. The outcome of this is normally reduced costs due to the economies of scale. Unlike the Beveridge model, the Bismarck Model emphasizes multi-funded healthcare, especially through an insurance payment that is normally agreed on between employers and their employees. In this healthcare payment system, there are a lot of competitive insurance markets. This can result in increased quality and effectiveness of healthcare supply, though it simultaneously promotes a lack of correspondence in access to healthcare services to beneficiaries due to different income levels and occupation types. In the Out-of-Pocket model, unlike the two models discussed above, there is no funding provided for individuals to access healthcare services. The patients in this model are expected to cater for their own healthcare needs and make direct payments from their own income. There is no formal system in place for this system, and it depends on what individuals can afford. For lower-income citizens, this is one of the biggest problems that leads to the high rate of inequities in the healthcare services. One of the major setbacks of this system is that it results in a lot of barriers in access to healthcare services.

These three different models provide valuable insights into how resources are managed and allocated to ensure efficiency. They also offer an opportunity for the designing of a synchronous strategy for operational healthcare forecasting. For instance, the emphasis of the Beveridge model on tax/government funding helps to provide inclusive data gathering and analysis for public health needs, and provide effective healthcare forecasting and allocation of resources. In addition to this, given that this model has a centralized structure, it can allow for a swift response to associated health crises, as well as to healthcare trends. In the case of the Bismarck model, valuable lessons can be provided from this model, due to its fragmented nature that provides extended knowledge regarding various private sector efficiencies and employer–employee relations. This can assist in providing solutions for integrating public health goals with private sector healthcare efficiencies. By collecting data from many insurance providers, this model is also effective in promoting forecasting efforts that can help to improve healthcare service delivery. Despite it being heavily criticized for promoting inequalities, the Out-of-Pocket model provides insights into the different market dynamics of healthcare services and the behaviors of patients. This is useful in providing mechanisms for demand forecasting, especially in a resource-limited situation.

A hybrid model approach is crucial to consider in efforts to combine these three models in designing of a healthcare framework for developing countries. Incorporating the Beveridge and Bismarck models would be useful in providing an effective system that provides universal access to healthcare while encouraging effectiveness through the participation of the private sector. For example, a healthcare system that is funded by the government and coupled with employer-employee contributions can result in balancing equity issues with the demand for high-quality healthcare services. In addition, it is noted that forecasting accuracy can be improved, and the identification of real-time resource needs and health trends can be enabled through leveraging data analytics from both the government and the private sector healthcare departments. Furthermore, insights from the Out-of-Pocket model can help in policy design regarding the reduction of financial barriers, and allow for the integration of low-income populations into high-quality healthcare delivery. Through the analysis of the healthcare models, this research highlights the pros and cons of each approach, and provides practical insights into a customized healthcare system that is helpful in addressing the unique needs of LMICs. The designed system model that combines all the elements of these healthcare models provides a comprehensive solution which balances healthcare access with efficiency and quality, while at the same time addressing equality issues. This synthesis aids this research’s purpose of improving healthcare forecasting through a systems thinking approach, combining both strategic and operational layers for the enhancement of forecasting and resource allocation.

### 5.1. Operational Layer: Analysis and Results

A first step in business process analysis is the mapping of the processes. This is critical in the operational layer, as it defines the execution process and segregates the steps, enabling the definition of requirements, such as the people, equipment and time for the execution of steps and the process. This definition of requirements, which are the data associated with the business process, can be used for analysis and optimization. This approach was adopted in prioritizing the activities of a hospital during periods of constrained energy supply in South Africa, and in the energy evaluation of COVID-19 measures of negative pressure systems and XP ultraviolet rays [69,70]. However, the application of business process mapping and simulation is not limited to the healthcare sector; this approach was also applied to a gold refinery in South Africa to determine the first-to-go business activities for digitalization in the business’s digital transformation journey. This approach was also applied to the services sector in South Africa to determine business tasks for optimization.

Business process mapping begins with the determination of the hierarchy that is most suitable to a business, as it is not possible to comprehensively capture all business activities and their associated interlinkages and cross-functionality via a single view [71]. The hierarchical structure is typically aligned with the organizational structure, but is focused on the systematic categorization of activities. In typical cases, a four-level hierarchical structure is appropriate to define the categorization of business activities. A four-level hierarchy for healthcare facilities is illustrated in Figure 6.

**Figure 11:**
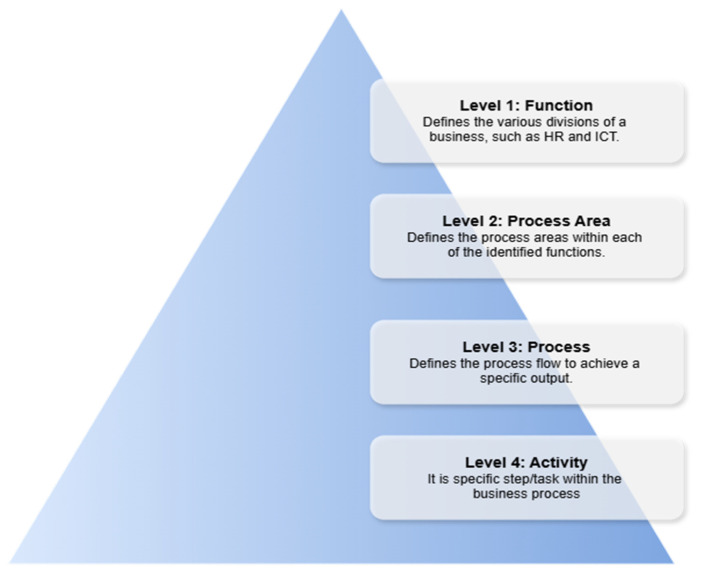
The proposed hierarchy for a healthcare facility.

This four-level hierarchy was applied to a hospital, with the levels illustrated in Figure 11.

**Figure 12:**
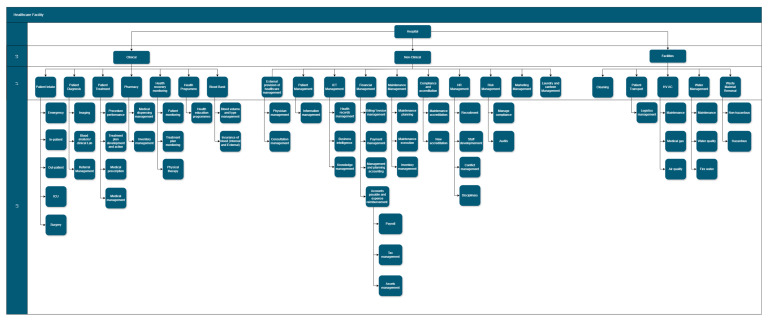
Structure of business processes.

Four-level business processes are typically not captured in a summary view, due to the level of detail. A sample of a four-level business process of a surgical process [69] is illustrated in Figure 12, with each step referred to as a business process step.

**Figure 13:**
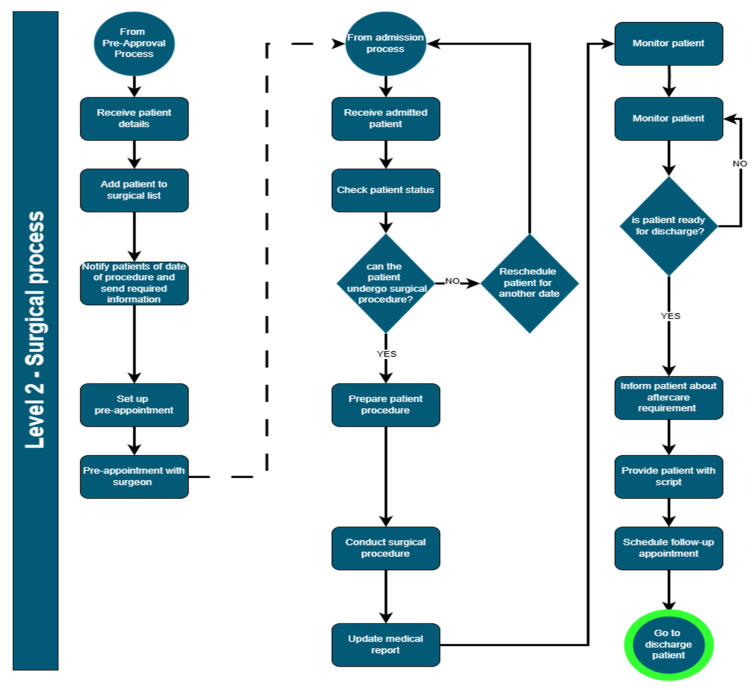
Business process sample.

Level 4 enables the quantification of data, such as equipment requirements and associated energy requirements, personnel requirements, time for execution and ICT requirements (including hardware and software). This is illustrated in Figure 13.

**Figure 14:**
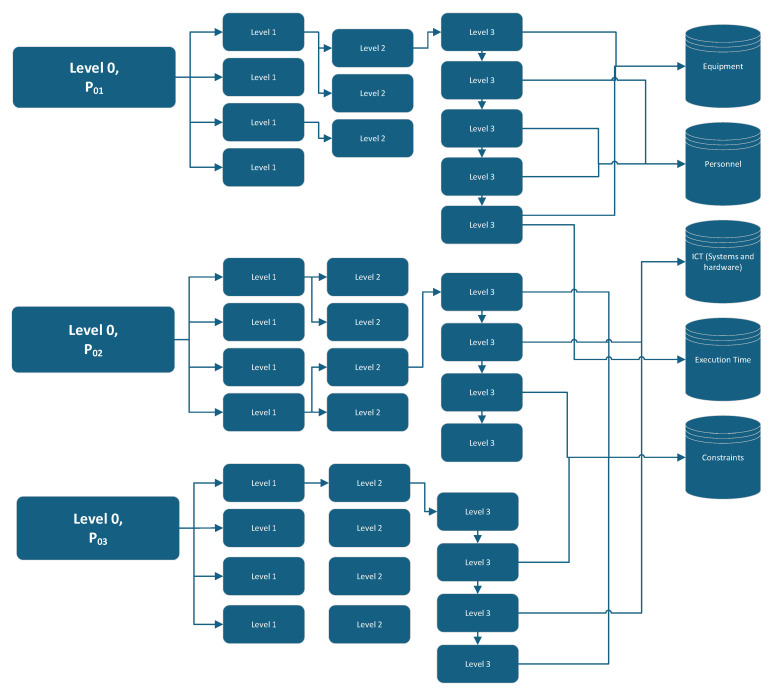
Process hierarchy.

Figure 14 illustrates that business processes are interconnected; the patient intake process is connected to the blood analysis/clinical lab process. There are modified processes for ICU, emergency and surgery, given the time sensitivity of these activities.

The data gathered at the business process level can be analyzed in various ways. A simple illustration of data utilization is illustrated in Table 1.

The data gathered for each business process enable an understanding of the actual operations of healthcare. This information has a dual purpose:

- It can be used in defining the optimum financial model for a healthcare sector.
- It would enable the user to quantifiably define the opportunities and challenges of the current financing model. It can thus be used to optimize the healthcare sector, within boundaries, based on the current financial approach.

### 5.2. Ecosystem Modeling

The ultimate objective is to build a simulation system that will simplify the process of understanding the entire healthcare system and every feature that is involved in improving healthcare performance. The healthcare system is too diverse and complex for one to fully understand all aspects of the system, but the approach taken simplifies the system from a complex level to a basic level. The results and discoveries from this study are a stepping stone to the involvement of advanced AI models that can simulate the healthcare system. The purpose of the simulation is to try to find the perfect-quality healthcare system model for all, that will be inclusive to affluent and deprived citizens, regardless of geolocation as well.

**Figure 15:**
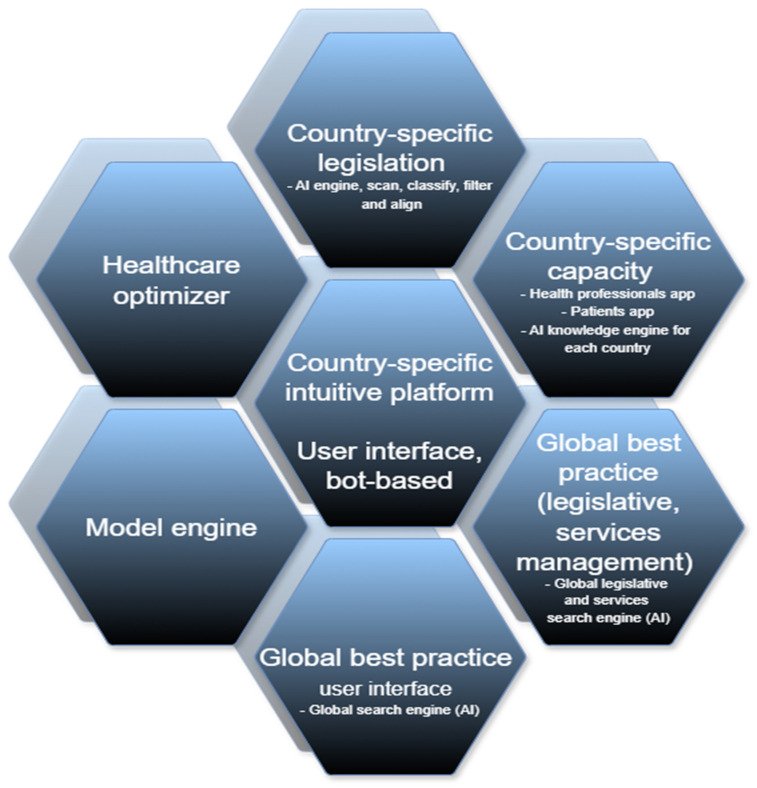
Healthcare optimizer.

The final outcome of this study is the design of the NHS system model for synchronous strategies in operational healthcare forecasting, as portrayed in Figure 15.

An ecosystem model must have the capacity to collect data and model/optimize the collective healthcare ecosystem. The key considerations are as follows:

- Legislative tracking
- Capacity tracking
- Global best practice
- Skills tracking
- A best practice optimizer
- A comprehensive process model
- A data-aligned AI decision engine
- Real-time data collection

## 6. Conclusions

The integration of the fundamental models discussed provides a solution for the design of an effective and equitable integrated NHS, especially in developing countries. This integrated system is crucial for a sustainable response and the delivery of healthcare services that cater for the population’s needs. This framework can provide guidelines on how to include universal coverage and private contributions. It is important for policymakers to provide an all-inclusive health data infrastructure that can foster forecasting and efficient strategic planning processes that include all affected stakeholders from various areas of the healthcare sector. Public health programs should be reinforced by targeted investments in health infrastructure and labor force development, advancing a resilient healthcare system that is able to meet the demands of an evolving population. This comprehensive approach not only addresses immediate health needs, but also provides the foundation for long-term health equity and sustainability. In conclusion, this study recognizes that the proposed healthcare optimizer platform remains conceptual, with limitations primarily centered around the boundaries of the model’s design. While the focus was on outlining its core framework, further exploration of implementation, scalability and resource requirements was not included, as these aspects were outside of the scope of this initial design phase. However, the authors acknowledge this limitation, and recommend that future research examine these factors in greater detail. Additionally, further studies should explore the broader contextual elements that could influence the platform’s effectiveness and its potential for real-world application.

## 7. Current Work

The research team envisage the development and testing of the above ecosystem model in multiple LMIC contexts, with data collection and the advancement of the ecosystems approach being developed in conjunction with three LMICs, including South Africa, India and Zimbabwe.

## Figures and Tables

**Table 1:** Data utilization and extraction for model.

Business Enterprise Function	Business Area (Level 1)	Business Process (Level 2)	Business Process Step (Level 3)	Resource Requirement	Resource Energy Demand (kW)	Resource Utilization Time (h)
Clinical	Patient Intake	In-patient or Out-patient or surgery	Create the order requisition for blood testing	Laptop	0.03	0.17
Clinical	Patient Intake	In-patient or Out-patient or surgery	Collect blood samples	Laptop	0.03	0.25
Clinical	Patient Intake	In-patient or Out-patient	Transfer blood samples for clinical testing	Manual	0	
Clinical	Patient Diagnosis	Blood analysis/clinical lab	Schedule testing	Laptop	0.03	0.07
Clinical	Patient Diagnosis	Blood analysis/clinical lab	Conduct testing	Blood analyzer	1.5	1
Clinical	Patient Diagnosis	Blood analysis/clinical lab	Capture results	Laptop	0.03	0.08
Clinical	Patient Diagnosis	Blood analysis/clinical lab	Approve results	Laptop	0.03	0.17
Clinical	Patient Diagnosis	Blood analysis/clinical lab	Send results to physician	Laptop	0.03	0.17

## Data Availability

The data presented in this study are available on request from the corresponding author.
